# Supplementary material for: Patterns of multimorbidity and risk of severe SARS-CoV-2 infection: an observational study in the U.K
Source: BMC Infect Dis. 2021 Sep 4;21:908. doi: 10.1186/s12879-021-06600-y (PMC8418288; doi:10.1186/s12879-021-06600-y)
Supplement: Supplementary file 1 — Additional file 1: Figure S1. Flow chart of participants included in the UK Biobank Study. Table S1. Literature search on the most common pre-existing comorbidities in patients with severe SARS-CoV-2 infection. Table S2. Association between multimorbidity index using 3 or more conditions and risk of severe SARS-CoV-2 infection. Table S3. Sensitivity analyses using vitamin D levels at follow-up and last recorded air pollution levels. Table S4. Sensitivity analyses considering time in the study and removing cardiorespiratory fitness and C-reactive protein from the model. Checklist S1. Strengthening the Reporting of Observational Studies in Epidemiology (STROBE). [file 12879_2021_6600_MOESM1_ESM.pdf]

Additional File

Patterns of Multimorbidity and Risk of Severe SARS-CoV-2 Infection: an observational study in the U.K.

Yogini V Chudasama, Francesco Zaccardi, Clare L Gillies, Cameron Razieh, Thomas Yates, David E Kloecker, Alex V Rowlands, Melanie J Davies, Nazrul Islam, Samuel Seidu, Nita G Forouhi* and Kamlesh Khunti *

* Joint senior authors

Table of Contents

Figure S1: Flow chart of participants included in the UK Biobank Study 1

Table S1: Literature search on the most common pre-existing comorbidities in patients with severe SARS-CoV-2 infection 2

Table S2. Association between multimorbidity index using 3 or more conditions and risk of severe SARS-CoV-2 infection 6

Table S3. Sensitivity analyses using vitamin D levels at follow-up and last recorded air pollution levels 7

Table S4. Sensitivity analyses considering time in the study and removing cardiorespiratory fitness and C-reactive protein from the model 8

Checklist S1. Strengthening the Reporting of Observational Studies in Epidemiology (STROBE) 9

Figure S1: Flow chart of participants included in the UK Biobank Study

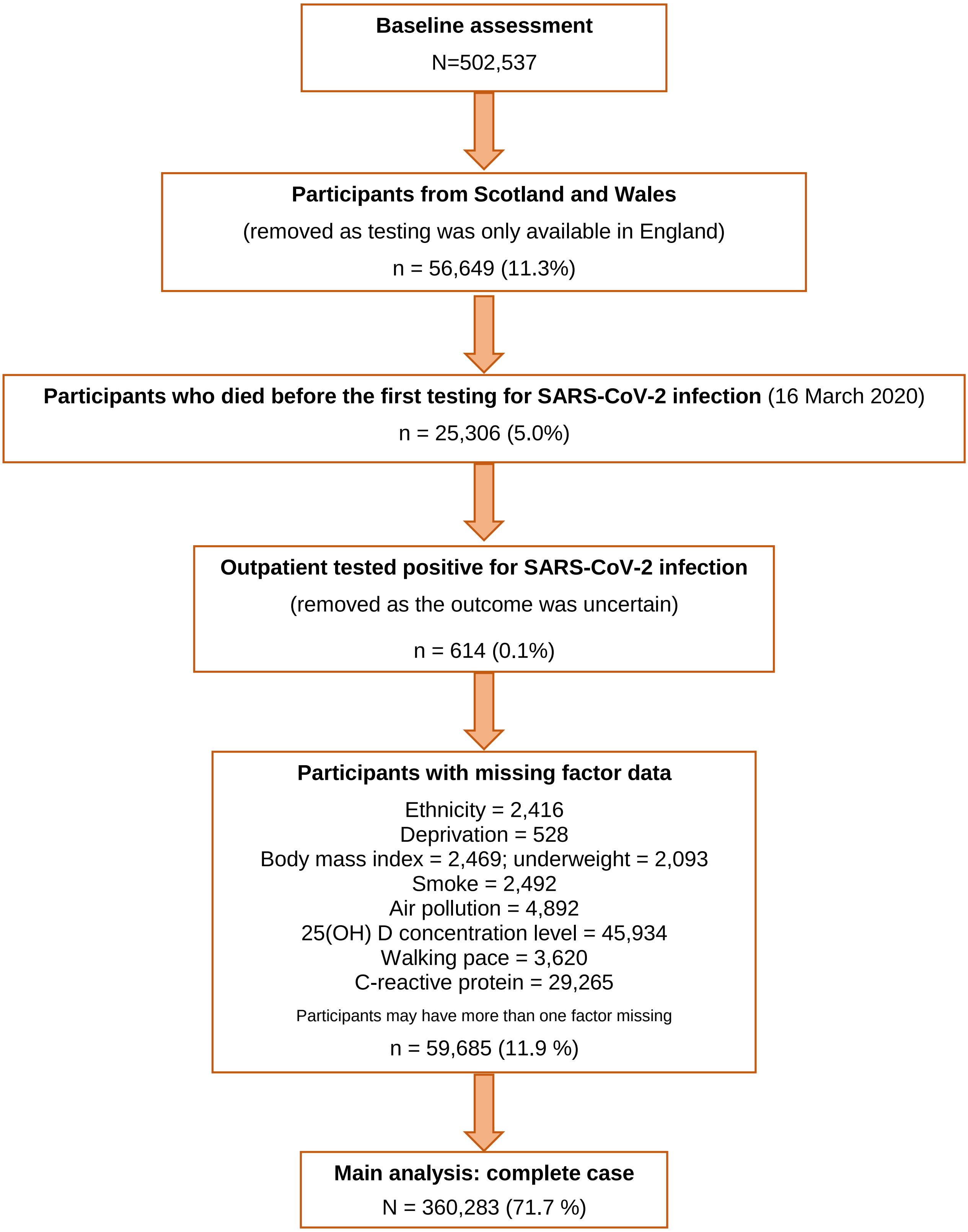

Table S1: Literature search on the most common pre-existing comorbidities in patients with severe SARS-CoV-2 infection

Reference	Type of study and date	Study population	Most common pre-existing comorbidities associated with patients with severe SARS-CoV-2 infection (hospitalised)
**Arentz et al, 2020 [1]**	Case series<br>February 20 to March 5, 2020	21 critically ill patients with COVID-19 in Washington State, United States	1. Chronic kidney disease<br>2. Heart failure<br>3. Diabetes<br>4. Chronic obstructive pulmonary disease<br>5. Obstructive sleep apnoea<br>6. Asthma
**Du et al. 2020 [2]**	Prospective cohort study,<br>25 December 2019, to 7 February 2020	179 patients who were hospitalised with COVID-19 to Wuhan Pulmonary Hospital, China	1. Hypertension<br>2. Diabetes mellitus<br>3. Cardiovascular or cerebrovascular diseases<br>4. Chronic digestive disorders<br>5. Tuberculosis<br>6. Cancer, malignancy<br>7. Peripheral vascular disease
**Emami et al. 2020 [3]**	Systematic review and meta-analysis<br>Until 15 February 2020<br>10 articles	3,403 hospitalised patients with COVID-19	1. Hypertension<br>2. Cardiovascular diseases<br>3. Diabetes mellitus<br>4. Chronic obstructive pulmonary disease<br>5. Cancer, malignancy<br>6. Chronic kidney disease
**Grasselli et al, 2020 [4]**	Retrospective case series<br>February 20 to March 18 2020	1591 critically ill patients admitted to ICUs in Lombardy, Italy	1. Hypertension<br>2. Cardiovascular disease<br>3. Hypercholesterolemia<br>4. Diabetes, type 2<br>5. Cancer, malignancy<br>6. Chronic obstructive pulmonary disease<br>7. Chronic liver disease<br>8. Chronic kidney disease
**Guan et al. 2020 [5]**	Retrospective case study,<br>11 December 2019, to 31 January 2020	1590 laboratory confirmed hospitalised patients from 575 hospitals in 31 provinces/autonomous regions/provincial municipalities across mainland China	1. Hypertension<br>2. Cardiovascular or cerebrovascular diseases<br>3. Diabetes mellitus<br>4. Hepatitis B infection<br>5. Chronic kidney disease<br>6. Cancer, malignancy

Reference	Type of study and date	Study population	Most common pre-existing comorbidities associated with patients with severe SARS-CoV-2 infection (hospitalised)
**Ji et al, 2020 [6]**	Nationwide retrospective case-control study<br>Until May 15 2020	Severe cases were 954 of 7,341, Korea	1. Hypertension<br>2. Diabetes mellitus<br>3. Chronic lower respiratory disease<br>4. Chronic renal failure
**Li X et al, 2020 [7]**	Retrospective study<br>January 26 to February 5 2020	548 patients as severe cases on admission, Tongji Hospital, Wuhan, China	1. Hypertension<br>2. Diabetes<br>3. Asthma<br>4. Coronary heart disease<br>5. Tuberculosis<br>6. Chronic obstructive pulmonary disease<br>7. Cancer, tumour<br>8. Chronic kidney disease<br>9. Hepatitis B
**Myers et al. 2020 [8]**	Retrospective cohort study,<br>March 1 2020, to March 31 2020	377 were treated as inpatients and 113 were treated in the ICU, in 21 hospitals, California, United States	1. Hypertension<br>2. Diabetes mellitus<br>3. Chronic kidney disease<br>4. Chronic obstructive pulmonary disease or asthma<br>5. Heart failure<br>6. Liver cirrhosis<br>7. Cancer, malignancy
**Petrilli et al, 2020 [9]**	Prospective cohort study<br>1 March 2020 and 8 April 2020	2741 were admitted to hospital, New York City and Long Island, United States	1. Hypertension<br>2. Cardiovascular disease<br>3. Asthma or chronic obstructive pulmonary disease<br>4. Diabetes<br>5. Cancer<br>6. Chronic kidney disease
**Q et al. 2020 [10]**	Retrospective cohort study,<br>January 30 2020, to February 11 2020	108 adult patients with COVID-19 were hospitalised in the Dabieshan Medical Center, Huanggang, China	1. Hypertension<br>2. Diabetes mellitus<br>3. Chronic obstructive pulmonary disease<br>4. Cardiovascular disease<br>5. Chronic liver disease<br>6. Cancer

**Reference**	**Type of study and date**	**Study population**	**Most common pre-existing comorbidities associated with patients with severe SARS-CoV-2 infection (hospitalised)**
**Richardson et al. 2020 [11]**	Case series,<br>March 1 2020, to April 4 2020	5,700 hospitalised patients with COVID-19, in 12 hospitals across New York, United States	1. Hypertension<br>2. Cardiovascular disease<br>3. Obesity<br>4. Diabetes mellitus<br>5. Cancer
**Yang J et al, 2020 [12]**	Systematic review and meta-analysis<br>Until 25 February 2020<br>7 articles	1,576 infected patients from hospitals in China	1. Hypertension<br>2. Diabetes mellitus<br>3. Respiratory system disease<br>4. Cardiovascular disease
**Yang X et al. 2020 [13]**	Retrospective study<br>Before 31 January 2020	52 critically ill adult patients with SARS-CoV-2 pneumonia who were admitted to the intensive care unit of Wuhan Jin Yin-tan hospital,<br>China	1. Cerebrovascular disease<br>2. Diabetes mellitus<br>3. Chronic cardiac disease<br>4. Chronic pulmonary disease
**Zhou et al. 2020 [14]**	Retrospective, multicentre cohort study<br>Before 31 January 2020	191 patients with COVID-19 (135 from Jinyintan Hospital and 56 from Wuhan Pulmonary Hospital), China	1. Hypertension<br>2. Diabetes mellitus<br>3. Coronary heart disease<br>4. Chronic obstructive lung disease<br>5. Cancer, Carcinoma<br>6. Chronic kidney disease

Google Scholar and PubMed searches for studies in published in English were carried out with the terms “comorbidity”; “severe SARS-CoV-2”; or “COVID-19 hospitalisation” on 3rd July 2020. In the table we reported the studies we deemed most relevant. We did not include studies that were already considered in the systematic reviews.

References

1. Arentz M YE, Klaff L, Lokhandwala S, Riedo FX, Chong M, Lee M: **Characteristics and Outcomes of 21 Critically Ill Patients With COVID-19 in Washington State**. *JAMA* 2020, **323**(16):1612-1614.
2. Du R-H, Liang L-R, Yang C-Q, Wang W, Cao T-Z, Li M, Guo G-Y, Du J, Zheng C-L, Zhu Q *et al*: **Predictors of mortality for patients with COVID-19 pneumonia caused by SARS-CoV-2: a prospective cohort study**. 2020.
3. Emami A, Javanmardi F, Pirbonyeh N, Akbari A: **Prevalence of Underlying Diseases in Hospitalized Patients with COVID-19: a Systematic Review and Meta-Analysis**. *Arch Acad Emerg Med* 2020, **8**(1).
4. Grasselli G ZA, Zanella A, Antonelli M, Cabrini L, Castelli A, Cereda D: **Baseline Characteristics and Outcomes of 1591 Patients Infected With SARS-CoV-2 Admitted to ICUs of the Lombardy Region, Italy**. *JAMA* 2020, **323**(16):1574-1581.
5. Guan W-j, Liang W-h, Zhao Y, Liang H-r, Chen Z-s, Li Y-m, Liu X-q, Chen R-c, Tang C-l, Wang T *et al*: **Comorbidity and its impact on 1590 patients with COVID-19 in China: a nationwide analysis**. 2020.
6. W J, K H, M K, J H, GH B, R L, Y N, H C, SY G, YH C *et al*: **Effect of Underlying Comorbidities on the Infection and Severity of COVID-19 in Korea: A Nationwide Case-Control Study**. *Journal of Korean medical science* 2020, **35**(25).
7. Li X XS, Yu M, Wang K, Tao Y, Zhou Y: **Risk factors for severity and mortality in adult COVID-19 inpatients in Wuhan | Elsevier Enhanced Reader**. *Journal of Allergy and Clinical Immunology* 2020, **146**(1):110-118.
8. Myers LC, The Permanente Medical Group KPNC, Oakland, Parodi SM, The Permanente Medical Group KPNC, Oakland, Escobar GJ, The Permanente Medical Group KPNC, Oakland, Liu VX, The Permanente Medical Group KPNC, Oakland: **Characteristics of Hospitalized Adults With COVID-19 in an Integrated Health Care System in California**. *JAMA* 2020, **323**(21):2195-2198.
9. Petrilli CM JS, Yang J, Rajagopalan H, O’Donnell L, Chernyak Y, Tobin KA, Cerfolio RJ: **Factors associated with hospital admission and critical illness among 5279 people with coronavirus disease 2019 in New York City: prospective cohort study**. 2020.
10. Q Y, P W, X W, G Q, M M, X T, X B, M D, W L, K L *et al*: **A retrospective study of risk factors for severe acute respiratory syndrome coronavirus 2 infections in hospitalized adult patients.** *Polish Archives of Internal Medicine* 2020, **130**(5):390-399.
11. Safiya Richardson JSH, Mangala Narasimhan, James M. Crawford, Thomas McGinn, Karina W. Davidson: **Presenting Characteristics, Comorbidities, and Outcomes Among 5700 Patients Hospitalized With COVID-19 in the New York City Area**. *JAMA* 2020, **323**(20):2052-2059.
12. Yang J, Zheng Y, Gou X, Pu K, Chen Z, Guo Q, Ji R, Wang H, Wang Y, Zhou Y: **Prevalence of comorbidities and its effects in coronavirus disease 2019 patients: A systematic review and meta-analysis**. *Int J Infect Dis* 2020, **94**:91-95.
13. X Y, Y Y, J X, H S, J X, H L, Y W, L Z, Z Y, M F *et al*: **Clinical Course and Outcomes of Critically Ill Patients With SARS-CoV-2 Pneumonia in Wuhan, China: A Single-Centered, Retrospective, Observational Study.** *The Lancet Respiratory medicine* 2020, **8**(5).
14. Zhou F YT, Du R, Fan G, Liu Y, Liu Z, et al: **Clinical course and risk factors for mortality of adult inpatients with COVID-19 in Wuhan, China: a retrospective cohort study**. *T he Lancet* 2020, **395**(10229):1054-1062.

Table S2. Association between multimorbidity index using 3 or more conditions and risk of severe SARS-CoV-2 infection

**Risk of severe SARS-CoV-2 infection** (hospitalisation or death)	**OR (95% CI)**	**P-value**
**Age at test**		
< **60 years** (n=83,269)	2.01 (0.73, 5.52)	0.790
≥ **60 years** (n=277,014)	2.00 (1.66, 2.42)	
**Sex**		
**Women** (n=195,571)	1.81 (1.28, 2.57)	0.106
**Men** (n=164,712)	2.03 (1.63, 2.53)	
**Ethnicity**		
**White** (n=340,619)	1.94 (1.60, 2.36)	0.330
**Non-white** (n=19,664)	2.75 (1.60, 4.72)	
**Deprivation**		
**Least deprived** (n=180,147)	2.65 (1.98, 3.56)	0.070
**Most deprived** (n=180,136)	1.72 (1.36, 2.18)	
**Body mass index**		
**Normal** (n=120,764)	1.13 (0.55, 2.30)	0.340
**Overweight** (n=153,914)	2.25 (1.67, 3.04)	
**Obese** (n=85,605)	2.05 (1.59, 2.62)	
**Smoke**		
**Never** (n=200,669)	2.06 (1.49, 2.83)	0.577
**Previous** (n=124,882)	2.00 (1.56, 2.56)	
**Current** (n=34,732)	1.71 (1.00, 2.92)	
**Air pollution (NO_2)**		
**Low/moderate level** (n=335,378)	2.00 (1.64, 2.43)	0.467
**High level** (n=24,905)	2.12 (1.25, 3.60)	
**25-hydroxyvitamin D levels**		
**Severe deficiency** (n=43,558)	1.22 (0.76, 1.95)	0.036
**Sufficient** (n=316,725)	2.24 (1.84, 2.74)	
**Cardiorespiratory fitness**		
**Slow walking pace** (n=25,569)	2.00 (1.51, 2.64)	0.772
**Steady-brisk walking pace** (n=334,714)	1.98 (1.55, 2.54)	
**C-reactive protein level**		
**Normal** (n=282,720)	2.24 (1.78 2.81)	0.121
**High** (n=77,563)	1.68 (1.23, 2.30)	

Odds ratios comparing subjects with multimorbidity (≥3 conditions) vs without multimorbidity (reference: <3 conditions). P-values tested for interaction.

OR=odds ratio; CI=confidence interval; NO_2=nitrogen dioxide.

Models adjusted for age at test, sex, ethnicity, deprivation, smoking status, body mass index, air pollution, 25-hydroxyvitamin D, cardiorespiratory fitness, C-reactive protein, season at blood draw, and regular intake of vitamin D supplement.

Table S3. Sensitivity analyses using vitamin D levels at follow-up and last recorded air pollution levels

**Risk of severe SARS-CoV-2 infection** (hospitalisation or death)	**OR (95% CI)**	
	**2 or more pre-existing multimorbidity index conditions**	**3 or more pre-existing multimorbidity index conditions**
**25-hydroxyvitamin D levels categories**		
**<25 nmol/L** (n=43,558)	1.80 (1.36, 2.37)	1.22 (0.76, 1.94)
**25-50 nmol/L** (n=148,624)	2.01 (1.69, 2.40)	2.09 (1.59, 2.75)
**50-75 nmol/L** (n=125,430)	1.84 (1.47, 2.30)	2.71 (1.94, 3.80)
**≥75 nmol/L** (n=42,671)	1.81 (1.27, 2.58)	1.76 (0.96, 3.23)
**25-hydroxyvitamin D levels at follow-up ***		
**Severe deficiency** (n=2,165)	2.30 (0.75, 7.11)	1.21 (0.14, 10.36)
**Sufficient** (n=12,376)	3.14 (1.67, 5.93)	2.92 (0.99, 8.56)
**Air pollution (NO_2) last recorded**		
**Low-moderate level** (n=344,059)	1.93 (1.71, 2.17)	2.08 (1.72, 2.52)
**High level** (n=16,224)	1.66 (1.03, 2.68)	1.25 (0.58, 2.69)
**Air pollution (PM 2.5) last recorded**		
**Low-moderate level** (n=192,392)	2.08 (1.75, 2.46)	2.50 (1.92, 3.25)
**High level** (n=167,891)	1.78 (1.51, 2.08)	1.68 (1.30, 2.18)

Odds ratios comparing subjects with multimorbidity vs without multimorbidity (reference).

OR=odds ratio; CI=confidence interval; NO_2=nitrogen dioxide.

Models adjusted for age at test, sex, ethnicity, deprivation, smoking status, body mass index, air pollution, 25-hydroxyvitamin D, cardiorespiratory fitness, C-reactive protein, season at blood draw, and regular intake of Vitamin D supplement.

***** Model adjusted for regular intake of Vitamin D supplement at follow-up, the season of blood draw was not known at follow-up.

Table S4. Sensitivity analyses considering time in the study and removing cardiorespiratory fitness and C-reactive protein from the model

**Risk of severe SARS-CoV-2 infection** (hospitalisation or death)	**OR (95% CI)**	
	**2 or more pre-existing multimorbidity index conditions**	**3 or more pre-existing multimorbidity index conditions**
**Additionally adjusted for time in the study ***	1.91 (1.70, 2.15)	2.02 (1.68, 2.43)
**Removal of cardiorespiratory fitness, C-reactive protein**	2.09 (1.87, 2.35)	2.40 (2.00, 2.87)

Odds ratios comparing subjects with multimorbidity vs without multimorbidity (reference).

OR=odds ratio; CI=confidence interval.

Unless stated the models adjusted for age at test, sex, ethnicity, deprivation, smoking status, body mass index, air pollution, 25-hydroxyvitamin D, cardiorespiratory fitness, C-reactive protein, season at blood draw, and regular intake of Vitamin D supplement.

* Time in the study was calculated from the date of the baseline characteristics collection to the date of hospitalisation of SARS-CoV-2, date of mortality or date of last censoring in the study.

Checklist S1. Strengthening the Reporting of Observational Studies in Epidemiology (STROBE)

	Item No	Recommendation	
**Title and abstract**	1	(*a*) Indicate the study's design with a commonly used term in the title or the abstract	Title and abstract
		(*b*) Provide in the abstract an informative and balanced summary of what was done and what was found	Abstract, methods and findings
**Introduction**			
Background/rationale	2	Explain the scientific background and rationale for the investigation being reported	Introduction paragraph 1-3
Objectives	3	State specific objectives, including any prespecified hypotheses	Introduction paragraph 4
**Methods**			
Study design	4	Present key elements of study design early in the paper	Methods, Study Population
Setting	5	Describe the setting, locations, and relevant dates, including periods of recruitment, exposure, follow-up, and data collection	Methods, Study Population
Participants	6	(*a*) *Cohort study*—Give the eligibility criteria, and the sources and methods of selection of participants. Describe methods of follow-up	Methods, Study Population
		(*b*) *Cohort study*—For matched studies, give matching criteria and number of exposed and unexposed	NA
Variables	7	Clearly define all outcomes, exposures, predictors, potential confounders, and effect modifiers. Give diagnostic criteria, if applicable	Methods, Multimorbidity index, Outcome measures, Effect modifiers
Data sources/ measurement	8*	For each variable of interest, give sources of data and details of methods of assessment (measurement). Describe comparability of assessment methods if there is more than one group	Methods, Multimorbidity index, Outcome measures, Effect modifiers
Bias	9	Describe any efforts to address potential sources of bias	Methods, Study Population, Statistical Analysis paragraph 3
Study size	10	Explain how the study size was arrived at	Supporting Information, Figure S1
Quantitative variables	11	Explain how quantitative variables were handled in the analyses. If applicable, describe which groupings were chosen and why	Methods, Multimorbidity index, Effect modifiers, Statistical analysis
Statistical methods	12	(*a*) Describe all statistical methods, including those used to control for confounding	Methods, Statistical analysis
		(*b*) Describe any methods used to examine subgroups and interactions	NA
		(*c*) Explain how missing data were addressed	Methods, Statistical analysis paragraph 1
		(*d*) *Cohort study*—If applicable, explain how loss to follow-up was addressed	NA
		(*e*) Describe any sensitivity analyses	Methods, Statistical analysis paragraph 3

**Results**			
Participants	13*	(a) Report numbers of individuals at each stage of study—e.g. numbers potentially eligible, examined for eligibility, confirmed eligible, included in the study, completing follow-up, and analysed	Methods, Study Population
		(b) Give reasons for non-participation at each stage	Methods, Study Population
		(c) Consider use of a flow diagram	Supporting Information, Figure S1
Descriptive data	14*	(a) Give characteristics of study participants (e.g. demographic, clinical, social) and information on exposures and potential confounders	Results, Participant Characteristics
		(b) Indicate number of participants with missing data for each variable of interest	Supporting Information Figure S1
		(c) *Cohort study*—Summarise follow-up time (e.g., average and total amount)	NA
Outcome data	15*	*Cohort study*—Report numbers of outcome events or summary measures over time	Results, Participant Characteristics, Pattern of multimorbidity
Main results	16	(*a*) Give unadjusted estimates and, if applicable, confounder-adjusted estimates and their precision (eg, 95% confidence interval). Make clear which confounders were adjusted for and why they were included	Results, Risk of severe SARS-CoV-2 infection
		(*b*) Report category boundaries when continuous variables were categorized	Results, Risk of severe SARS-CoV-2 infection
		(*c*) If relevant, consider translating estimates of relative risk into absolute risk for a meaningful time period	NA
Other analyses	17	Report other analyses done—e.g. analyses of subgroups and interactions, and sensitivity analyses	Results, Risk of severe SARS-CoV-2 infection, paragraph 2
**Discussion**			
Key results	18	Summarise key results with reference to study objectives	Discussion paragraph 1
Limitations	19	Discuss limitations of the study, taking into account sources of potential bias or imprecision. Discuss both direction and magnitude of any potential bias	Discussion paragraph 5
Interpretation	20	Give a cautious overall interpretation of results considering objectives, limitations, multiplicity of analyses, results from similar studies, and other relevant evidence	Discussion paragraph 2-5
Generalisability	21	Discuss the generalisability (external validity) of the study results	Discussion paragraph 6
**Other information**			
Funding	22	Give the source of funding and the role of the funders for the present study and, if applicable, for the original study on which the present article is based.	End of the manuscript
